# Exocyst-Dependent Membrane Addition Is Required for Anaphase Cell Elongation and Cytokinesis in *Drosophila*

**DOI:** 10.1371/journal.pgen.1005632

**Published:** 2015-11-03

**Authors:** Maria Grazia Giansanti, Timothy E. Vanderleest, Cayla E. Jewett, Stefano Sechi, Anna Frappaolo, Lacramioara Fabian, Carmen C. Robinett, Julie A. Brill, Dinah Loerke, Margaret T. Fuller, J. Todd Blankenship

**Affiliations:** 1 Istituto di Biologia e Patologia Molecolari del CNR, Dipartimento di Biologia e Biotecnologie, Università Sapienza di Roma, Roma, Italy; 2 Department of Physics, University of Denver, Denver, Colorado, United States of America; 3 Department of Biological Sciences, University of Denver, Denver, Colorado, United States of America; 4 Cell Biology Program, The Hospital for Sick Children, Toronto, Ontario, Canada; 5 Janelia Research Campus, Howard Hughes Medical Institute, Ashburn, Virginia, United States of America; 6 Department of Molecular Genetics, University of Toronto, Toronto, Ontario, Canada; 7 Department of Developmental Biology, Stanford University School of Medicine, Stanford, California, United States of America; University of Colorado, UNITED STATES

## Abstract

Mitotic and cytokinetic processes harness cell machinery to drive chromosomal segregation and the physical separation of dividing cells. Here, we investigate the functional requirements for exocyst complex function during cell division *in vivo*, and demonstrate a common mechanism that directs anaphase cell elongation and cleavage furrow progression during cell division. We show that *onion rings (onr)* and *funnel cakes (fun)* encode the *Drosophila* homologs of the Exo84 and Sec8 exocyst subunits, respectively. In *onr* and *fun* mutant cells, contractile ring proteins are recruited to the equatorial region of dividing spermatocytes. However, cytokinesis is disrupted early in furrow ingression, leading to cytokinesis failure. We use high temporal and spatial resolution confocal imaging with automated computational analysis to quantitatively compare wild-type versus *onr* and *fun* mutant cells. These results demonstrate that anaphase cell elongation is grossly disrupted in cells that are compromised in exocyst complex function. Additionally, we observe that the increase in cell surface area in wild type peaks a few minutes into cytokinesis, and that *onr* and *fun* mutant cells have a greatly reduced rate of surface area growth specifically during cell division. Analysis by transmission electron microscopy reveals a massive build-up of cytoplasmic astral membrane and loss of normal Golgi architecture in *onr* and *fun* spermatocytes, suggesting that exocyst complex is required for proper vesicular trafficking through these compartments. Moreover, recruitment of the small GTPase Rab11 and the PITP Giotto to the cleavage site depends on wild-type function of the exocyst subunits Exo84 and Sec8. Finally, we show that the exocyst subunit Sec5 coimmunoprecipitates with Rab11. Our results are consistent with the exocyst complex mediating an essential, coordinated increase in cell surface area that potentiates anaphase cell elongation and cleavage furrow ingression.

## Author Summary

The cell shape changes that underlie cell division are some of the most fundamental changes in cell morphology. Here, we show that a common membrane trafficking pathway is required for both the cell lengthening that occurs during anaphase, and the physical separation of a cell into two equal daughter cells. We measure and define the periods of surface area increase during cell division in *Drosophila* male germline cells, and demonstrate that subunits of the exocyst tethering complex are required for this process. Invagination of the cleavage furrow fails at an early stage in exocyst mutant spermatocytes, suggesting that membrane addition is part of the initial ingression mechanism. In the absence of exocyst complex function, vesicular trafficking pathways are disrupted, leading to enlarged cytoplasmic membrane stores, and disruption of Golgi architecture. In addition, a vesicular Rab protein, Rab11, biochemically associates with the exocyst complex subunit Sec5. These results suggest that remodeling of the plasma membrane and targeted increases in surface area are an active part of the fundamental mechanisms that permit eukaryotic cell division to occur.

## Introduction

Cytokinesis results in the physical separation of two daughter cells. Immediately prior to the initiation of cytokinesis, cells also begin to elongate along the spindle axis, concomitant with the anaphase spindle elongation that helps drive chromosomal separation. To achieve such a fundamental remodeling of shape and topology, cells martial multiple cytoskeletal and membrane trafficking pathways. Contraction of an equatorial actomyosin ring is required for inward progression of the cleavage furrow, and a further abscission process operates to fully separate the incipient daughter cells into two distinct membranous structures. In addition, processes that regulate membrane trafficking events are also required for successful cytokinesis [1–3].

Previous studies demonstrated that *Drosophila* male meiotic cells represent a sensitive system for identification of cellular components that contribute to cytokinesis [4]. Genes that regulate central spindle function, contractile ring assembly, phosphoinositide composition, and exocytic trafficking have all been identified through mutations that disrupt male germline cytokinesis. Trafficking proteins that are required for cytokinesis include the Conserved Oligomeric Golgi Complex complex (COG) subunits Cog5 and Cog7, the Rab11 GTPase, the Syntaxin 5 ER-to-Golgi vesicle-docking protein, the endosomal Arf6 GTPase, the phosphatidylinositol 4-kinase IIIβ Four Wheel Drive (Fwd), the TRAPPII complex subunit Brunelleschi, and phosphatidylinositol 4-phosphate [PI(4)P] effector GOLPH3 [5–14]. However, the final proteins in these exocytic pathways that may direct membrane addition at the cell surface have remained unidentified.

Spatial specificity of vesicle trafficking occurs through the targeting of exocytic vesicles at defined membrane sites by tethering complexes such as the exocyst complex [15,16]. The eight subunits of the exocyst (Sec3, Sec5, Sec6, Sec8, Sec10, Sec15, Exo70, and Exo84) were originally identified based on their role in polarized secretion in *Saccharomyces cerevisiae* [17] and were subsequently shown to form a complex that is highly conserved from yeast to mammals [18–23]. We have previously demonstrated that the Exo84 subunit of the exocyst complex mediates apical epithelial identity in *Drosophila* [24]. Other groups have shown that members of the *Drosophila* exocyst are required for membrane addition and expansion in developing oocytes and neurons, in photoreceptor cells and during embryonic cellularization [25–31]. Additionally, the exocyst complex has been shown to be required for cell abscission at the end of cytokinesis in mammalian tissue culture cells [32–35].

Here, we demonstrate that *funnel cakes (fun)* and *onion rings (onr)* encode the exocyst proteins Sec8 and Exo84, respectively. We show that dividing spermatocytes mutant for either *onr* or *fun* display an exceptionally early defect in progression of the cleavage furrow and fail to accumulate Rab11 and Giotto/Vibrator at the cell midzone. Quantitative analysis suggests that rather than disrupting gross membrane addition to the cell surface, these mutations specifically affect a trafficking pathway required for both anaphase cell elongation and cleavage furrow ingression.

## Results

### *fun* and *onr* encode *Drosophila* homologs of exocyst complex subunits

**Fig 1:**
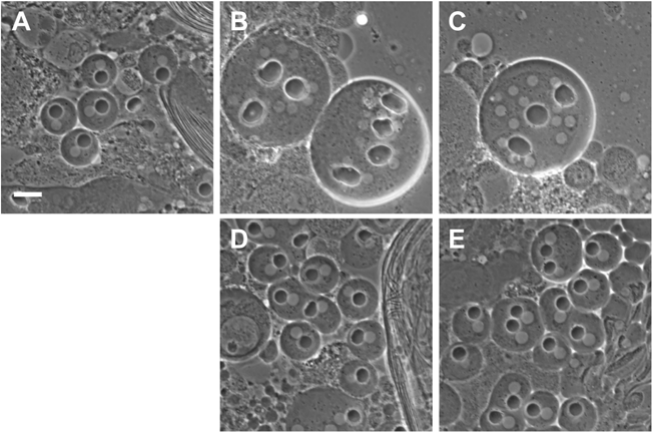
Rescue of *funnel cakes* and *onion rings* mutant cells by Sec8 and Exo84. (A) Phase contrast microscopy of wild type, *fun* ^*z1010*^/*Df(3R)Exel6145* mutant (B) and *onr* ^*z4840*^ */Df(3R)Espl3* mutant (C) male germline cells. In *fun* and *onr* mutant cells, cell division fails and multiple nuclei (white spherical objects) are observed in association with enlarged nebenkern (black spherical objects). In wild-type cells, single nuclei are found in association with nebenkern of approximately equal size. A single copy of a transgene containing either genomic Sec8 (D) or genomic Exo84 (E) rescues cytokinesis defects in *fun* ^*z1010*^/*Df(3R)Exel6145* (D) and *onr* ^*z4840*^ */Df(3R)Espl3* (E) mutant cells. Scale bar, 10 μm.

*fun* and *onr* were identified in a screen for mutations that disrupt cytokinetic events in male germline cells [4]. Previous characterization of *fun* and *onr* revealed that these mutations do not affect central spindle or F-actin ring formation in dividing spermatocytes. Nonetheless, in *fun* and *onr* mutants, cytokinesis fails at an early stage [4]. The *fun* ^*z1010*^ mutation was mapped to the 83C1;83C4 interval on chromosome III in the region of the *Sec8* gene. Deficiency mapping revealed that *fun* ^*z1010*^ failed to complement *Df(3R)Exel6145* for the male sterility and cytokinesis defects (Fig 1A and 1B). Two lines of evidence indicate that *fun* ^*z1010*^ is an allele of *Drosophila Sec8*, which encodes a protein with 35% identity to human and mouse Sec8 proteins and 19% identity to the *S*. *cerevisiae* Sec8 protein (S1 Fig). First, a 6.6 kb genomic transgene containing the predicted *Sec8* coding region, 1.0 kb of upstream promoter sequence, and 1.9 kb of downstream sequence fully rescued the cytokinesis defects in *fun* mutant male germline cells (Fig 1B and 1D). Indeed, 100% of onion-stage spermatids from *fun* ^*z1010*^/*Df(3R)Exel6145* males bearing a single copy of the rescuing transgene possess a wild type 1:1 ratio of nuclei to nebenkern (n = 102), compared with 0.8% in males of identical genotype devoid of the transgene (n = 125). Additionally, DNA sequencing of the *Sec8* gene in *fun* ^*z1010*^ mutant males revealed a C to T mutation resulting in replacement of a conserved Serine residue by Phenylalanine at position 322 of the predicted 985 amino acid polypeptide (S1 Fig). Together, these results provide clear evidence that *fun* ^*z1010*^ represents a mutation in the *Sec8* gene.

Remarkably, while *fun* ^*z1010*^ disrupted functioning of the Sec8 exocyst subunit, the *onr* mutation from the same phenotypic class of mutants [4] was previously shown to affect the Exo84 exocyst subunit [24]. In short, the *onr* ^*z4840*^ allele possesses a nonsense mutation that is predicted to generate a truncated protein containing 581 of 672 amino acids [24]. Consistent with this, a 4.5 kb genomic transgene containing the predicted *Exo84* coding region, 1.5 kb of upstream promoter sequence, and 1 kb of downstream sequence fully rescued cytokinesis defects in *onr* mutant male germline cells (Fig 1C and 1E; 98.2% of onion-stage spermatids from *onr* ^*z4840*^ */Df(3R)Espl3* hemizygous males bearing a single copy of the rescuing transgene exhibit a wild-type 1:1 ratio of nuclei to nebenkern (n = 112), compared to 0% in *onr* hemizygotes devoid of the transgene (n = 101).

### Localization of exocyst complex proteins in dividing spermatocytes

**Fig 2:**
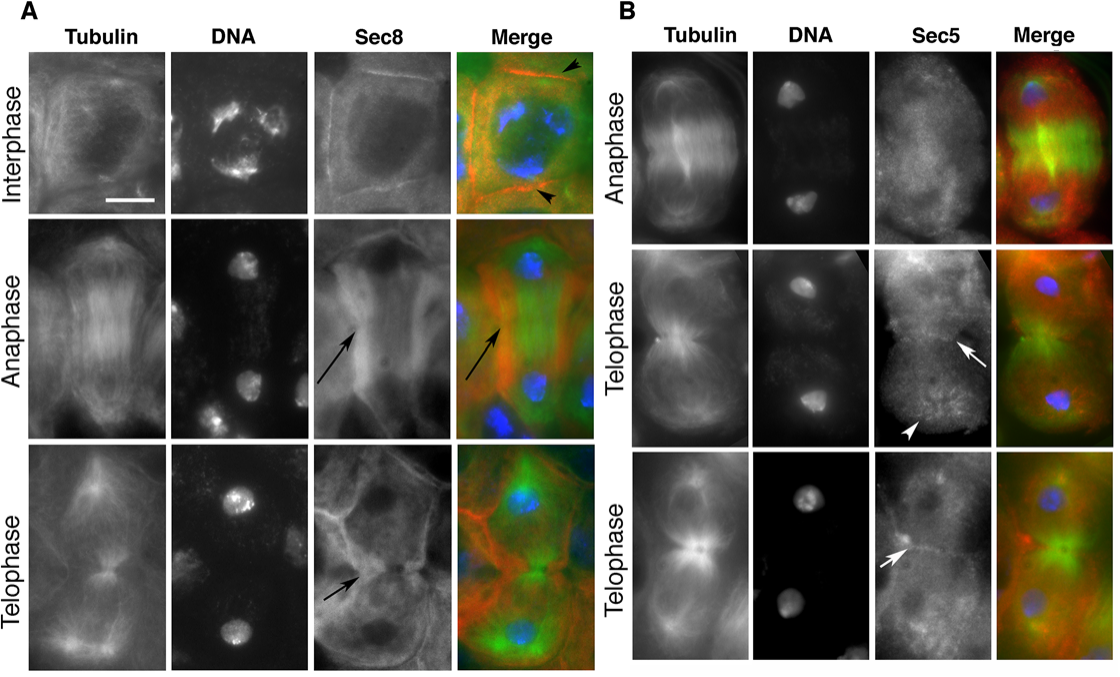
Localization of exocyst complex proteins in dividing spermatocytes. (A) Localization of Sec8 protein in wild-type primary spermatocytes. Interphase and dividing spermatocytes were stained for Tubulin (green), Sec8 (red) and DNA (blue). During interphase, Sec8 was mostly diffuse in the cytoplasm and enriched at the plasma membrane (arrowheads). In dividing spermatocytes, Sec8 appeared enriched in a broad cortical band that encircled the midzone (arrows) and was excluded from the poles. (B) Localization of Sec5 protein in wild-type dividing spermatocytes. Primary spermatocytes were stained for Tubulin (green), Sec5 (red) and DNA (blue). Note the enrichment of Sec5 in puncta at the astral microtubules (arrowhead) and at the cleavage furrow (arrows). Scale bar, 10 μm.

Localization of Sec8 protein was analyzed in primary spermatocytes from larval testes fixed with either methanol-free formaldehyde (Fig 2A) or methanol/formaldehyde (S2 Fig). Staining of interphase primary spermatocytes with anti-Tubulin and anti-Sec8 antibodies revealed that Sec8 protein was diffuse throughout the cytoplasm and enriched at the plasma membrane (Fig 2A). In dividing spermatocytes, in addition to localization at the plasma membrane, Sec8 was enriched in a broad cortical area at the cell equator and excluded from the poles (Fig 2A). During mid-telophase and late telophase, Sec8 protein accumulated at the cortex, near the ingressing furrow membrane (Fig 2A). Analysis of larval testes from transgenic animals expressing a GFP-Exo84 fusion protein revealed that, similar to Sec8, Exo84 appeared diffuse in the cytoplasm during interphase and became enriched in the furrow region during early telophase (S3 Fig). Analysis of dividing cells stained for Tubulin and *Drosophila* Sec5 revealed that Sec5 was enriched in small puncta at the astral microtubules and concentrated at the furrow region in telophase (Fig 2B).

### *onion rings* and *funnel cakes* mutant spermatocytes exhibit an early defect in the progression of cytokinesis

**Fig 3:**
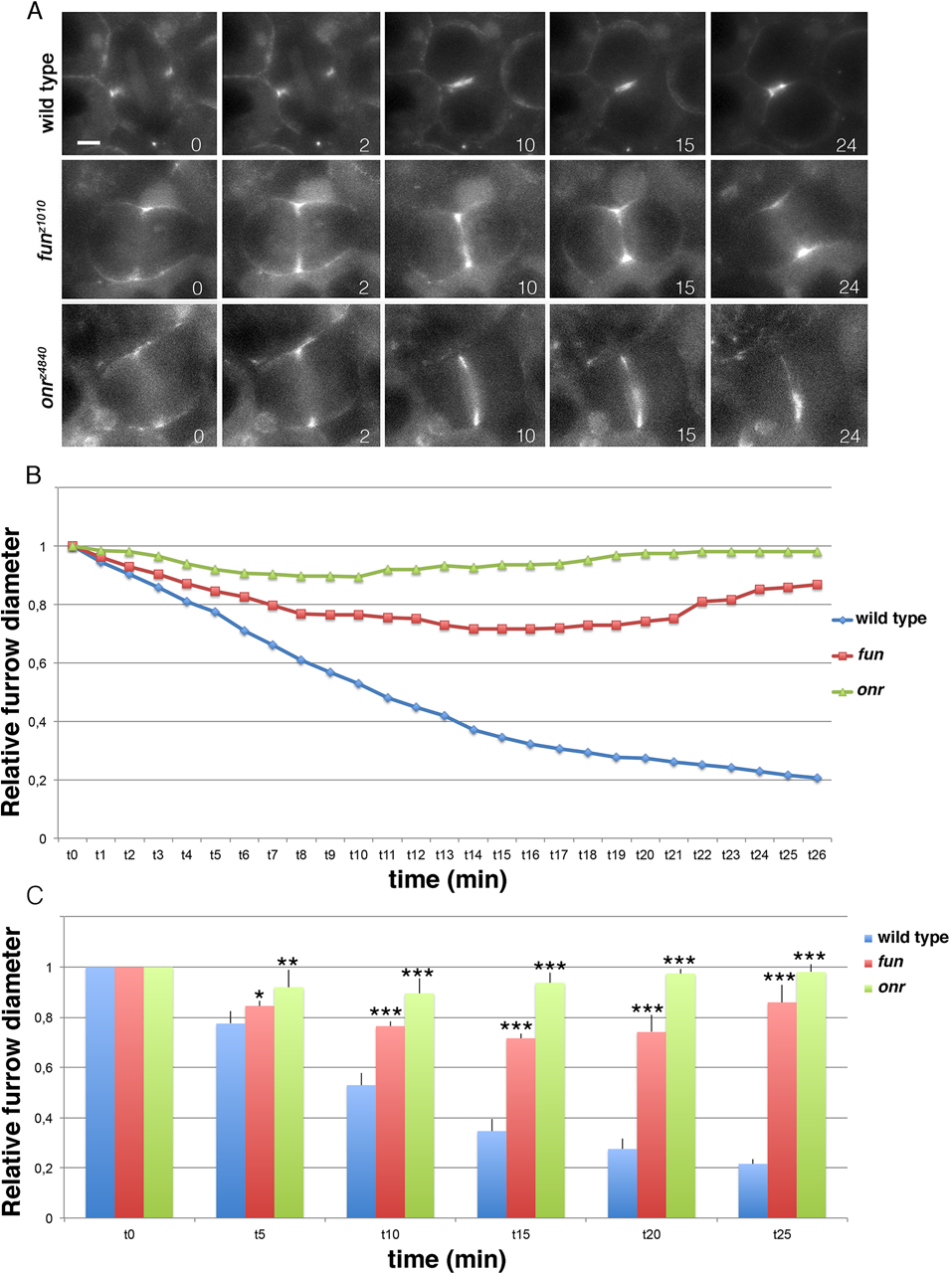
Defective cytokinetic ring ingression in *fun* and *onr* mutant cells. (A) Selected still frames from supplemental **S1, S2**and **S3**Movies. Dividing spermatocytes expressing the regulatory light chain of non-muscle myosin II, Sqh-GFP, were imaged starting from the beginning of anaphase. Numbers at the bottom of each frame indicate minutes from the beginning of imaging. Note that the Sqh-GFP ring undergoes minimal constriction (*fun*) or fails to constrict (*onr*) in mutant cells. Scale bar, 10μm. (B) Dynamics of cleavage furrows in *fun* and *onr* mutants. Furrow diameters (relative to the diameter at t = 0) in dividing spermatocytes from wild type, *fun* ^*z1010*^ */Df(3R)Exel6145* (*fun*) and *onr* ^*z4840*^ */Df(3R)Espl1 (onr)* males expressing Sqh-GFP and undergoing ana-telophase were plotted over time. (C) Furrow diameters (relative to the diameter at time = 0) were plotted at 5-minute intervals. Furrow diameters were measured in movies from dividing spermatocytes expressing Sqh-GFP and undergoing ana-telophases (n = 9 wild type, n = 8 *fun* and n = 8 *onr)*. Error bars indicate standard deviations. *p = 0.0035, **p = 0.0008;***p = 0.0001, significantly different from control in the Student t test.

Previous data showed that *onr* and *fun* mutations exhibited normal F-actin ring formation and central spindle assembly in dividing spermatocytes [4]. However, in mid to late telophase spermatocytes from *onr* and *fun* mutants, F-actin rings appeared poorly constricted and the central spindles were less dense than in wild type. Imaging of wild-type primary spermatocytes expressing myosin II regulatory light chain fused to GFP (Sqh-GFP, [5]) revealed that dividing spermatocytes (n = 9) assembled Sqh-GFP rings during anaphase that underwent full constriction within 20 minutes (Fig 3A and 3B; S1 Movie). In contrast, in dividing spermatocytes from either *fun* ^*z1010*^ */Df(3R)Exel6145* (n = 8) or *onr* ^*z4840*^ */Df(3R)Espl1* (n = 8), Sqh-GFP rings underwent minimal constriction accompanied by furrow regression and contractile ring rupture during the time of observation (Fig 3A and 3B and S2 Movie).

### Cell elongation, cytokinesis and expansion of cell surface area are defective in *onr* and *fun* mutant cells

**Fig 4:**
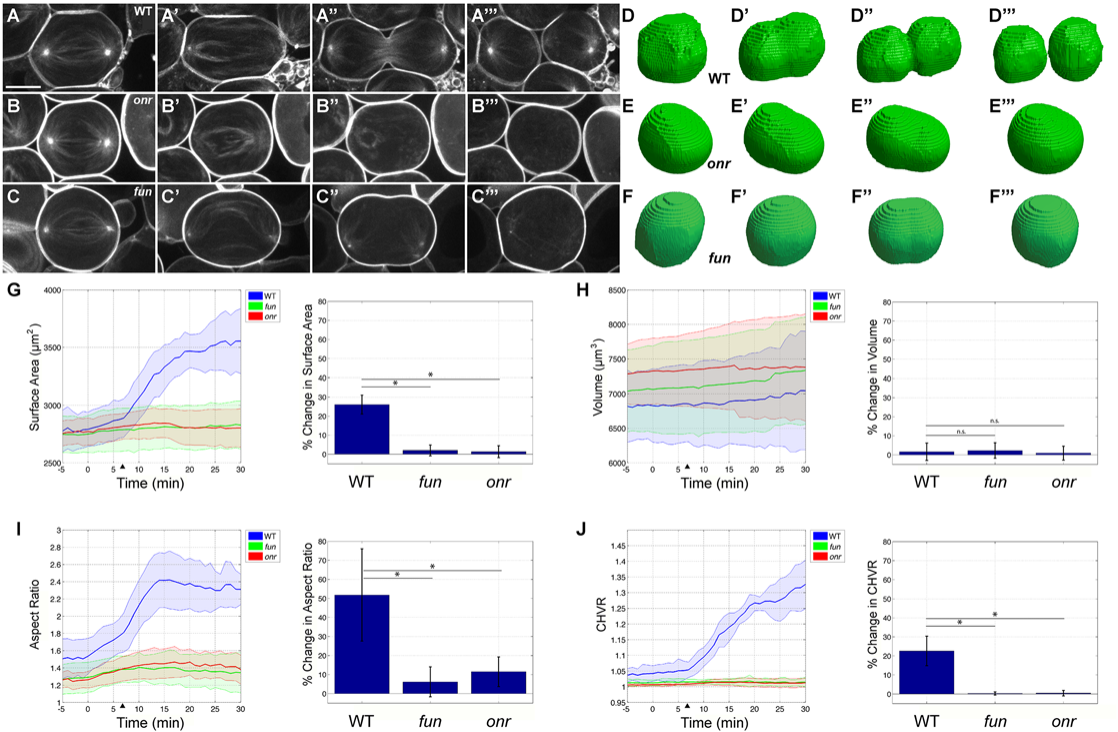
Failure in anaphase elongation, cleavage furrow progression, and surface area addition in *onr* and *fun* mutant cells. (A-C) Still frames from time-lapse confocal microscopy of wild-type (A), *onr* ^*z4840*^mutant (B), and *fun* ^*z1010*^ mutant (C) male germline cells expressing PLCδd-PH-GFP and β-Tub-GFP (imaged simultaneously in single channel). Cells are shown just prior to elongation (A, B, C), immediately before ingression (A’, B’, C’), during ingression (A”, B”, C”), and after successfully completing or failing to complete cytokinesis (A”‘, B”‘, C”‘). (D-F) Representative segmented and voxelized cells of wild-type (D), *onr* ^*z4840*^ mutant (E), and *fun* ^*z1010*^ mutant (F) cells. (G-J) Quantitative computational analysis of surface area (G), volume (H), aspect ratio (I), and convex hull volume ratio (a measurement of furrow ingression, J) in wild-type (blue), *onr* ^*z4840*^ mutant (red), and *fun* ^*z1010*^ mutant (green) cells. Left, lines are average values of wild-type (n = 8), *onr* (n = 11), and *fun* (n = 10) segmented cells. Data from individual cells were aligned such that t = 0 is the start of anaphase elongation, while arrowheads mark the initiation of cytokinesis in wild-type cells (see Materials and Methods). Right, quantitation of percent change from t = 0 to t = 25 min. Increases in surface area, aspect ratio, and CHVR observed in wild-type cells are disrupted in *onr* ^*z4840*^ and *fun* ^*z1010*^ mutant cells, while no significant difference is observed in volume. Prior to the start of anaphase elongation, cell volume and surface area were nearly identical in wild type, *onr* mutant, and *fun* mutant cells (p-values ranging from 0.0838 to 0.5969). All movies start during early anaphase and end after successful (wild-type) or failed (*fun* and *onr*) cytokinesis. Shaded region indicates standard error (G-J); *p<0.0001, significantly different from control in the two-sample Student t-test; n.s. = not significant, p>0.23. Scale bar, 10 μm.

In examining *onr* and *fun* mutant cells, we observed that dividing spermatocytes did not appear to lengthen along the spindle axis as much as wild-type cells do prior to cytokinesis. This elongation during anaphase may identify a time when a critical increase in surface area is initiated. To examine this quantitatively in an unbiased fashion, we developed a computational approach to segment cell boundaries and volumes. Dividing primary spermatocytes from wild-type and mutant males expressing PLCδ-PH-GFP [37], a plasma membrane marker, and β-Tub-GFP [38], a spindle and microtubule marker, were imaged by spinning disc microscopy (Fig 4A–4C). Image sets were acquired with XY resolutions of 0.166 microns per pixel and a Z-layer spacing of 1 micron every 60 seconds. Cells were then segmented using an automated 3D seeded watershed algorithm (Fig 4D–4F; S4–S6 Movies). From these voxelized representations of the cells, we computed a number of parameters that describe cellular geometries as male germline cells divide. Cell volume was computed as the sum of the voxel volumes, while surface area was computed as the sum of the areas of the exposed voxel surfaces.

To quantify ingression of the furrow, we used the convex hull volume ratio (CHVR). For a set of points in 3D space, the convex hull is the smallest convex spatial body spanned by a subset of the points that contains all the points of the set, i.e., the smallest convex envelope. The CHVR is defined as the convex hull volume divided by the actual segmented volume (schematically depicted in S4F Fig). By definition, the convex hull volume can be greater or equal to the actual volume, greater when concavities are present and equal when fully convex. Thus for an ellipsoid or sphere the CHVR = 1. For an idealized example of two perfect equal sized spheres touching at a point the CHVR = 1.25. Therefore, the CHVR provides a quantitative global measure of the amount of ingression.

The behaviors of wild-type cells were very consistent (Fig 4G–4J). Wild-type volume did not change significantly during cytokinesis (Fig 4H, p = 0.5297 when comparing wild-type cells at t = 0 to t = 25 min). Cytokinesis is therefore dependent on an increase in surface area. For the idealized geometry of a sphere dividing into two spheres of half the volume, the increase in surface area is approximately 26%. Our wild-type data are in good agreement with this percentage increase (26.1%), and the peak rate of increase is approximately 63 μm^2^/min. The average aspect ratio increased by just over 51.8%, and the average CHVR increased by 23% over the course of 25 minutes (Fig 4I and 4J). In contrast, *onr* ^*z4840*^ mutant cells had a brief period where surface area temporarily increased at a peak rate of 5.0 μm^2^/min, and surface area increased by 1.3% over 25 minutes (Fig 4G). In *fun* ^*z1010*^ mutant cells, the peak rate of surface area increase was 3.0 μm^2^/min, a rate similar to *onr* mutants but over 20 times slower than wild type, and the total percent increase over 25 minutes was 2.0% (Fig 4G). Intriguingly, cell volume and surface area were nearly identical in wild type, *onr* mutant, and *fun* mutant cells prior to the start cell division, suggesting that there is not a general blockade of plasma membrane trafficking in *onr* and *fun* mutants (Fig 4G and 4H). This also further suggests that directed trafficking specifically during anaphase cell elongation and cytokinesis may be an essential mediator of cell shape change.

An essential requirement for *onr* and *fun* function during anaphase cell elongation and cytokinesis can also be observed by directly examining the aspect ratio and the CHVR in these two mutants. In both mutants, the aspect ratio initially displayed a slight increase but peaked at 1.5 in *onr* ^*z4840*^ mutants and at 1.4 in *fun* ^*z1010*^ mutants before it then started to decline (as compared to 2.4 in wild-type cells). Similarly, cleavage furrow progression was disrupted in *onr* and *fun* mutant cells. Intriguingly, ingression of the cleavage furrow failed almost immediately in spermatocytes lacking *onr* or *fun* function (Fig 4B”, 4C” and 4J). During this process, the average CHVR reached a peak of 1.015 in *onr* mutants. Thus, on average, the volume of the ingression furrow was at most 1.5% of the cell volume. In *fun* mutant cells the CHVR peaked at 1.018. These results suggest that, *in vivo*, Exo84 and Sec8 function is required for a core set of cell shape changes that occur during cell division.

### *onr* and *fun* disrupt the Golgi compartments in Drosophila spermatocytes

**Fig 5:**
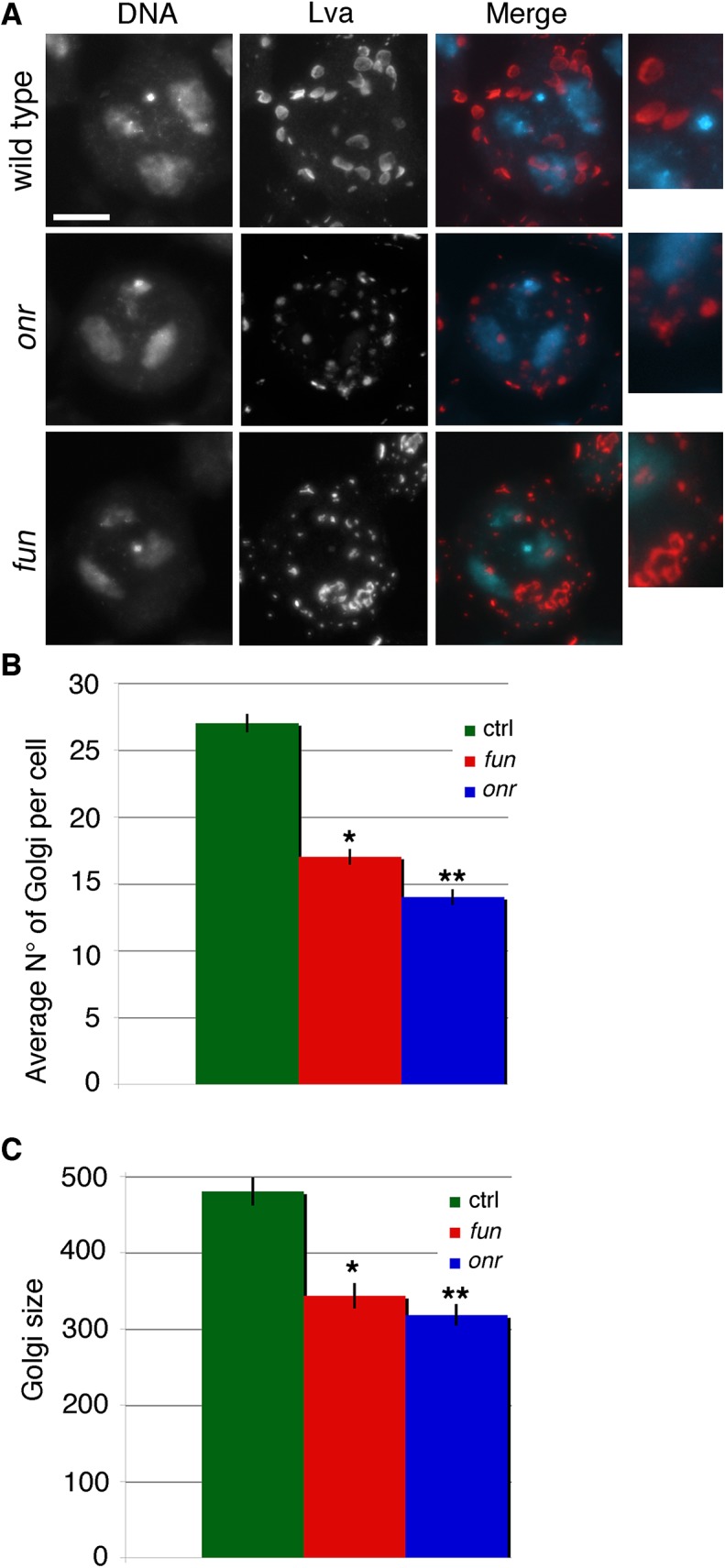
Defects in Golgi structure in *fun* and *onr* mutant cells. (A) G2 primary spermatocytes from wild-type, *onr* ^*z4840*^ */Df(3R)Espl3* and *fun* ^*z1010*^/*Df(3R)Exel6145* mutant males, stained for the Golgin Lva (red) and DNA (blue). Enlargements of Golgi stacks are shown on the right of each panel. Scale bar, 10 μm. (B) Average number of Golgi bodies per cell (± SEM) visualized in G2 spermatocytes from wild type (n = 50), *onr* ^*z4840*^ */Df(3R)Espl3 (onr*, n = 48), or *fun* ^*z1010*^/*Df(3R)Exel614* (*fun*, N = 48) after staining for Lva. Numbers of Golgi per cell in *fun* and *onr* mutants are significantly different from wild type in the Student t test:*p<0.0001, **p<0.0001. (C) Average area (± SEM) of Golgi bodies, quantified by ImageJ (expressed in arbitrary units), in G2 primary spermatocytes stained for Lva, Golgi sizes are significantly different in *fun* ^*z1010*^/*Df(3R)Exel614* (*fun*) and *onr* ^*z4840*^ */Df(3R)Espl3 (onr)* compared to wild type using the Student t test, *p<0.0001, **p<0.0001.

Several mutations in membrane trafficking components have been shown to disrupt the structure and/or the number of Golgi stacks in interphase primary spermatocytes [12,13,39]. To test whether *onr* and *fun* are required for Golgi organization in these cells, we used the Golgin Lava lamp (Lva) as a marker to examine the structure and distribution of the Golgi by immunofluorescence [40]. This analysis revealed defects in both the size and the number of Golgi stacks in *onr* and *fun* mutants (Fig 5A and 5B).

**Fig 6:**
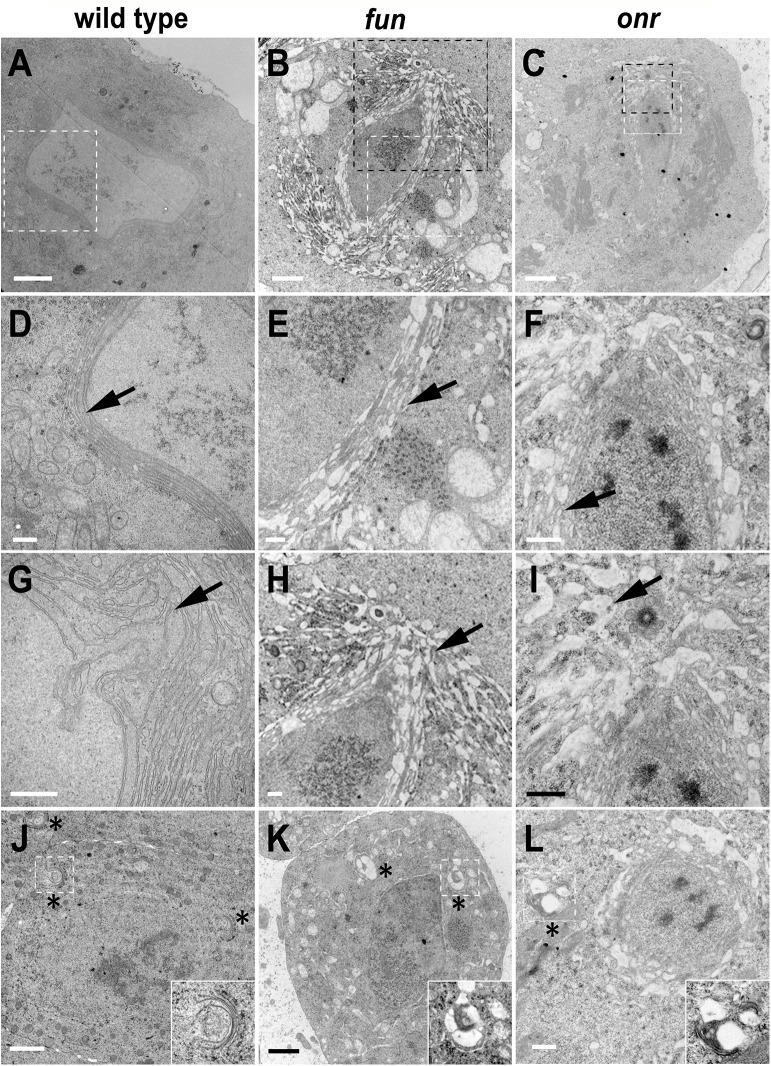
Defects in morphology and ultrastructure of parafusorial membranes and Golgi bodies in *fun* and *onr* mutant cells. Transmission electron micrographs showing parafusorial membranes (A-F), astral membranes (G-I), and Golgi bodies (J-L) in *fun* and *onr* mutant spermatocytes. Parafusorial and astral membranes (arrows) are enlarged, fragmented and vacuolated in *fun* ^*z1010*^/*Df(3R)Exel6145* (B, E, H) and *onr* ^*z4840*^ */Df(3R)Espl3* (C, F, I) dividing spermatocytes. (D, E, F) panels are magnified images of areas surrounded by white squares in (A, B, C). (H, I) panels are magnified images of areas surrounded by black squares in (B, C). Golgi bodies (asterisks) show vacuolated regions in *fun* (K) and *onr* (L) mutant spermatocytes. Golgi bodies surrounded by white squares in (J-L) are magnified in insets. Scale bars are 2 μm (A-C, J, K) or 500 nm (D-I, L).

Since surface area addition was defective in *onr* and *fun* mutant cells, and Golgi architecture was also disrupted, we analyzed the ultrastructure of spermatocyte cells by transmission electron microscopy (TEM) to determine if internal membrane compartments are altered. Intriguingly, *onr* and *fun* mutant cells displayed large accumulations of cytoplasmic membranes (Fig 6B and 6C). Indeed, parafusorial and astral membranes appeared enlarged, fragmented and vacuolated in *fun* and *onr* mutant dividing spermatocytes (Fig 6B and 6C, 6E–6F, 6H and 6I). Additionally, Golgi compartments were bloated and vacuolated when *fun* (10/14 Golgi bodies, or 71%) or *onr* (10/10 Golgi bodies, or 100%) functions were disrupted (Fig 6K and 6L), as compared to wild type (1/15 Golgi bodies, or 7%). Moreover, the extent of cisternal stacking within the Golgi was vastly reduced and the cisternae appeared disrupted by the vacuolated regions, potentially explaining the apparent fragmentation of the Lva signal in *fun* and *onr* mutant spermatocytes. Additionally, as Lva marks cis Golgi compartments [7,40], these results suggest that the expansion and bloating may preferentially affect medial or trans Golgi compartments. These results are consistent with a failure in vesicle trafficking to the cell surface required to mediate cell remodeling and elongation during anaphase and cytokinesis.

### Defects in Rab11 localization in *onr* and *fun* mutant spermatocytes

**Fig 7:**
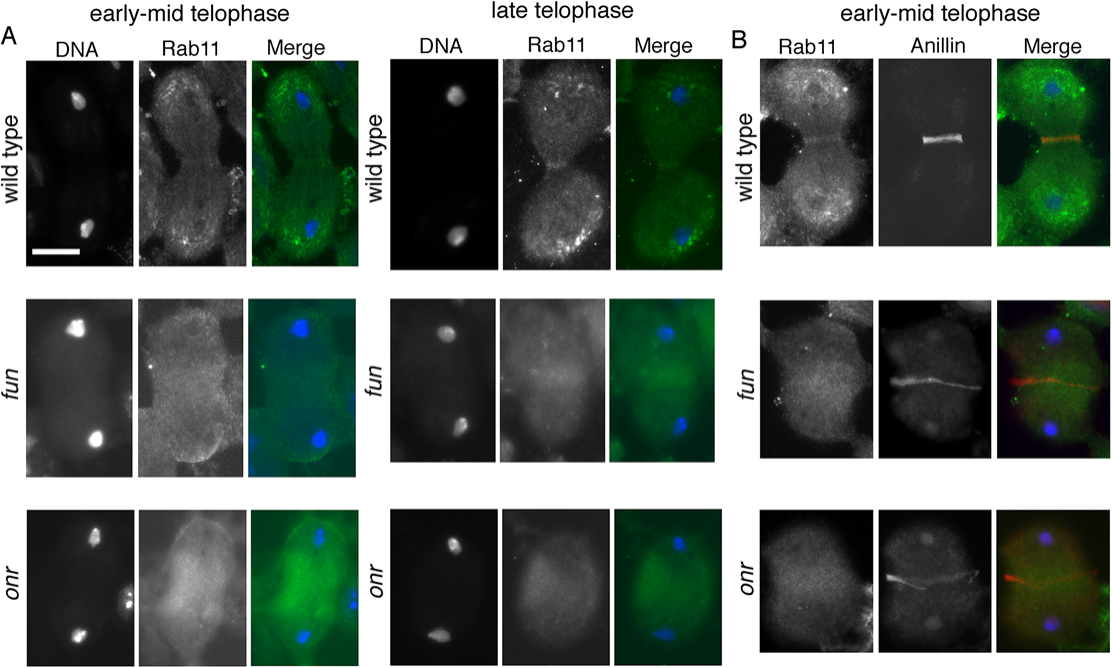
*onr and fun mutations* disrupt localization of Rab11 protein in dividing spermatocytes. (A) Telophase spermatocytes from wild type, *fun* ^*z1010*^/*Df(3R)Exel6145 (fun)* and *onr* ^*z4840*^ */Df(3R)Espl3 (onr)* stained for Rab11 (green) and DNA (blue). (B) Telophase spermatocytes from wild type, *fun* ^*z1010*^/*Df(3R)Exel6145 (fun)* and *onr* ^*z4840*^ */Df(3R)Espl3 (onr)* stained for Rab11 (green), Anillin (red) and DNA (blue). Scale bar, 10 μm.

**Fig 8:**
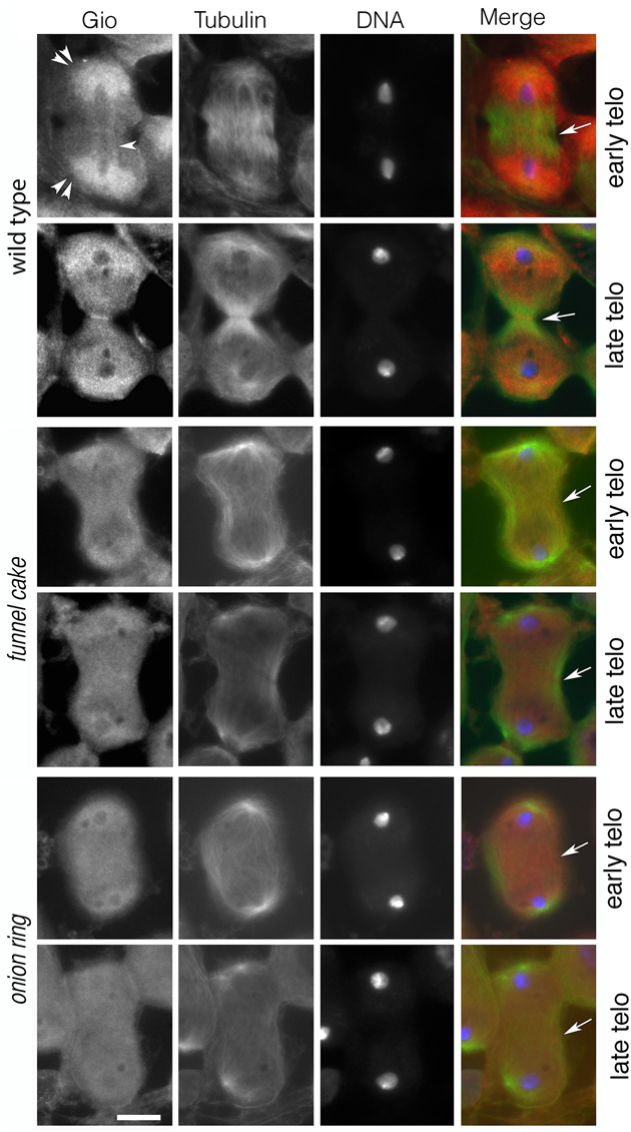
The PITP Giotto fails to concentrate at the midzone of dividing spermatocytes from *fun* and *onr* males. Spermatocytes were stained with anti-Tubulin (green), anti-Gio (red) and DAPI (blue). Arrows indicate the cleavage site. Wild type, *fun* ^*z1010*^/*Df(3R)Exel6145*, and *onr* ^*z4840*^ */Df(3R)Espl3* were stained for Tubulin (green), Gio (red) and DNA (Blue). Double arrowheads point to astral membranes, arrowheads indicate parafusorial membranes. Scale bar, 10 μm.

As Rab11 has been shown to be essential for cytokinesis during male meiotic divisions [10], we examined Rab11 behaviors in cells in which exocyst function has been compromised. Rab11 localization was abnormal in *fun* and *onr* mutant dividing spermatocytes (Fig 7A). In wild type, Rab11 was enriched in puncta at the cell poles during anaphase and telophase (n = 38; Fig 7A) and accumulated at the cleavage furrow during mid-telophase. By contrast, in ana-telophase spermatocytes from *fun* ^*z1010*^/*Df(3R)Exel6145* mutants, Rab11 was enriched in few puncta at the cell poles and failed to concentrate into a tight band at the midzone (Fig 7A). In all the telophase cells from *fun* ^*z1010*^/*Df(3R)Exel6145* mutants (n = 30; Fig 7A), Rab11 appeared enriched in a broad midzone area. Localization of Rab11 in *onr* ^*z4840*^ */Df(3R)Espl3* dividing spermatocytes also appeared diffuse at the midzone and excluded from the cell poles (n = 27; Fig 7A and 7B). Localization of Rab11 was also examined in dividing spermatocytes simultaneously stained for Rab11 and the furrow membrane marker anillin (Fig 7B). In wild-type telophase cells, Rab11 and anillin colocalized at the cleavage furrow (n = 32). In telophase cells from both *onr* ^*z4840*^ */Df(3R)Espl3* (n = 28) and *fun* ^*z1010*^/*Df(3R)Exel6145* (n = 24) mutants, anillin and Rab11 failed to co-localize at the equatorial cortex (Fig 7B). Rather, anillin formed a large ring at the equatorial cortex, consistent with defects in contractile ring constriction, and Rab11 accumulated at the midzone. In addition, *onr* and *fun* were also required for normal localization of phosphatidylinositol transfer protein Giotto/Vibrator (Gio/Vib, [9,12,41]; Fig 8). In wild-type anaphase and early telophase spermatocytes, Gio was enriched at the endoplasmic reticulum (ER) derived membranes that comprise the astral and parafusioral membrane arrays (Fig 8, [41]). In wild-type early (n = 23) and late telophases (n = 30), Gio also concentrated at the cleavage furrow (Fig 8). In early telophases from *fun* ^*z1010*^/*Df(3R)Exel6145* (n = 24) and *onr* ^*z4840*^ */Df(3R)Espl3* (n = 28) mutants, Gio was diffuse throughout the cells and failed to accumulate to the astral and parafusioral membrane arrays or to the cleavage furrow (Fig 8). Gio localization remained diffuse in late telophases from *fun* (n = 26) and *onr* (n = 28), (Fig 8).

### *onr* and *fun* interact with *Rab11*

**Fig 9:**
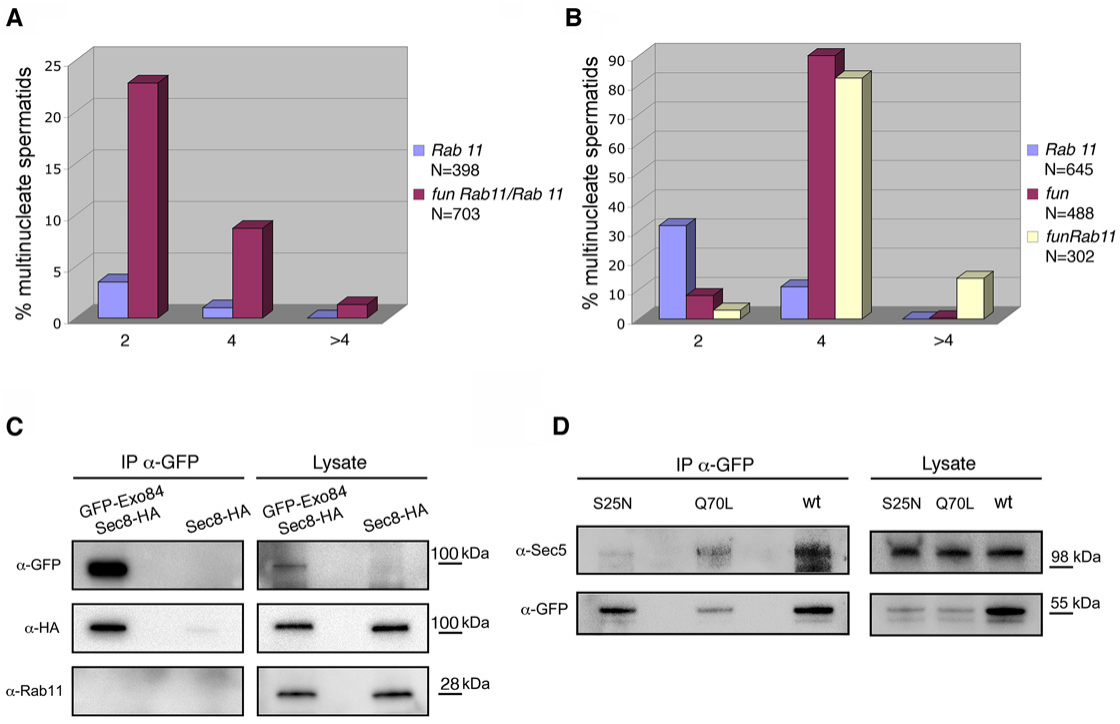
*onr* and *fun* mutations interact with mutations in *Rab11*. (A) Frequencies of early spermatids containing 2, 4 or more than 4 nuclei per nebenkern in testes from either *Rab11* ^*93Bi*^ */Rab11* ^*93Bi*^ *(Rab11) or fun* ^*z1010*^ *Rab11* ^*93Bi*^ */+ Rab11* ^*93Bi*^ *(fun Rab11/Rab11)* mutant males. (B) Frequencies of early spermatids containing multiple nuclei (2, 4 or more than 4 nuclei) per nebenkern in testes from either *Rab11* ^*93Bi*^/*Rab11* ^*E(To)3*^ *(Rab11)*, *fun* ^*z1010*^ */fun* ^*z1010*^ *(fun)*, or *fun* ^*z1010*^ *Rab11* ^*93Bi*^ *fun* ^*z1010*^ *Rab11* ^*E(To)3*^ *(fun Rab11)* mutant males. (C) Co-IP of HA-Sec8 with GFP-Exo84. Protein extracts from testes expressing either HA-Sec8 and GFP-Exo84 or HA-Sec8 alone were immunoprecipitated with anti-GFP (i.e., GFP-trap beads) and immunoblotted for either GFP, HA or Rab11. (D) Co-IP of Sec5 with YFP-Rab11. Protein extracts from testes expressing either wild-type YFP-Rab11 (wt), YFP-Rab11^Q70L^ (Q70L) or YFP-Rab11^S25N^ (S25N) were immunoprecipitated for YFP (using GFP-trap beads) and blotted for either YFP or Sec5.

The *onr* and *fun* mutants interacted genetically with *Rab11* mutants. Heterozygosity for *fun* dramatically increased the frequency of cytokinesis failures caused by homozygosity for the weak *Rab11* allele *Rab11* ^*93Bi*^, indicating a strong genetic interaction. *fun* ^*z1010*^ *Rab11* ^*93Bi*^ */+Rab11* ^*93Bi*^ males raised at 25°C exhibited a 7-fold increase in the percentage of multinucleate spermatids relative to testes from *Rab11* ^*93Bi*^ */Rab11* ^*93Bi*^ single mutants (Fig 9A and 9B). In addition, although *Rab11* ^*93Bi*^ and *Rab11* ^*93Bi*^/*Rab11* ^*E(To)11*^ transheterozygotes were viable, as were *fun* ^*z1010*^ */ fun* ^*z1010*^ flies, *fun* ^*z1010*^ *Rab11* ^*93Bi*^ */ fun* ^*z1010*^ *Rab11* ^*E(To)11*^ double mutants died mostly at early larval stages. Examination of testes from rare escaper larvae of genotype *fun* ^*z1010*^ *Rab11* ^*93Bi*^ */ fun* ^*z1010*^ *Rab11* ^*E(To)11*^ revealed that 13.9% of spermatids exhibited more than four nuclei per mitochondrial derivative, indicating a dramatic increase in cytokinesis failures during the gonial divisions that precede meiosis (Fig 9B). *Rab11* also interacted genetically with *onr*. *onr* ^*z4840*^ *Rab11* ^*93Bi*^ double mutants died in early larval stages, as did individuals that were homozygous for *onr* ^*z4840*^ and transheterozygous for *Rab11* ^*93Bi*^/*Rab11* ^*E(To)11*^.

To test whether Rab11 associated with the exocyst complex proteins Sec8 and Exo84 encoded by *onr* and *fun*, we performed co-immunoprecipitation (Co-IP) experiments using testis extracts. Immunoprecipitation by GFP-trap revealed that Sec8-HA co-precipitated with GFP-Exo84, consistent with the two proteins being subunits of the exocyst complex. Although we did not detect Rab11 in the precipitates from lysates of testes expressing GFP-Exo84, we could demonstrate biochemical interaction between Rab11 and Sec5 when YFP-Rab11 proteins expressed in adult testes, were immunoprecipitated with antibodies against GFP (Fig 9E). Sec5 co-immunoprecipitated with both YFP- tagged wild type Rab11 and Rab11^Q70L^ proteins, but only weakly with Rab11^S25N^.

## Discussion

The evolutionarily conserved octameric exocyst complex has been proposed to tether exocytic vesicles to specific sites on the plasma membrane and to regulate the SNARE complex during vesicle fusion [17,42,43]. A role for the exocyst in cell division was originally described in both budding and fission yeast where the exocyst proteins localize at the cleavage site and are required for vesicle trafficking during cytokinesis [19,44]. Here we provide evidence that the exocyst complex is required for the major cell shape changes that occur in dividing animal cells during anaphase and telophase. Through automated computational analysis of live *Drosophila* spermatocytes, we have shown that membrane addition correlates specifically with onset of anaphase cell elongation and that membrane addition peaks during early stages of cytokinetic furrow ingression in wild-type cells. Spermatocytes carrying mutations in the Exo84 or Sec8 proteins display a greatly reduced rate of surface area growth specifically at anaphase and cytokinesis, indicating a requirement for exocyst complex function in guiding plasma membrane expansion and remodeling in dividing cells. In agreement with this hypothesis, TEM analysis of *onr* and *fun* spermatocytes showed a massive build up of cytoplasmic astral membranes in dividing cells and altered Golgi architecture in interphase primary spermatocytes, suggesting that defective vesicular trafficking through these membrane compartments may result in reduced membrane material for the surface area increase required during anaphase cell elongation and cytokinesis. Indeed, proper localization of the Rab11 GTPase and PITP Gio to the cleavage site required wild-type Exo84 and Sec8 function.

In cultured mammalian cells, the exocyst is required late in cytokinesis for final resolution of the intercellular bridge [32–34], yet Sec5 and Exo84 are enriched in the cleavage furrow during early telophase [45]. Our data provide evidence for an early requirement for the exocyst during cytokinesis. Time-lapse analysis of spermatocytes undergoing anaphase and telophase showed that *fun* and *onr* mutations did not prevent recruitment of myosin II light chain at the cell equator. However, the Sqh rings assembled in the exocyst mutants underwent minimal or no constriction and failed to mediate cleavage furrow invagination. This is consistent with our previous characterization of *fun* and *onr* mutants, which revealed defects in F-actin ring contraction [4]. Failure to assemble functional contractile rings accompanied by early cleavage furrow regression also characterize *Drosophila* mutants in other vesicle trafficking components, including the COG complex subunits Cog5 and Cog7 [7,12], the ortholog of the yeast TRAPP II (trafficking transport protein particle II) TRS120p subunit [11], the PI4K Fwd [5], the Arf6 and Rab11GTPases [8,10], and GOLPH3 [13]. Defects in myosin II rings and incomplete furrow ingression were also observed in *Dyctiostelium discoideum* clathrin null cells [46]. Additionally *Drosophila* S2 cells depleted of syntaxin 1 displayed defective actin rings [47]. These observations suggest the existence of a close interplay between contractile ring dynamics and membrane trafficking at the cleavage furrow [7,10,11,48]. It has been proposed that altered membrane addition at the cleavage furrow would impair plasma membrane remodeling at the furrow and physically obstruct the contraction of the actomyosin ring [9,10]. In addition, transport of exocytic vesicles and their fusion with the furrow membrane might also be necessary to target structural components of the contractile apparatus or factors that regulate its constriction. In agreement with this, live imaging of actin and endocytic vesicles in cellularizing *Drosophila* embryos has suggested a model in which F-actin and vesicles are transported as a unit to the furrow site as F-actin-associated vesicles [49].

Interestingly, several studies have reported that Rab11 protein binds to two distinct exocyst complex subunits, Sec5 and Sec15 [29,50–53]. We have shown that Sec5 coimmunoprecipitates with Rab11 from *Drosophila* testis extracts, suggesting that these proteins may form a complex in spermatocytes. Furthermore, we have demonstrated that subcellular localization of Rab11 protein depends on *onr* and *fun* and that *Rab11* genetically interacts with both *onr* and *fun*. Remarkably, immunofluorescent analysis of telophase spermatocytes from *fun* mutants revealed that Rab11 accumulated in a broad cortical area, suggesting that Rab11-containing vesicles failed to reach the cleavage furrow plasma membrane. Together, these results indicate that exocyst complex proteins cooperate with the Rab11 GTPase in directing vesicle trafficking required for proper cytokinesis. In agreement with this idea, ultrasensitive live-imaging of fluorescently-tagged Sec8 in cultured mammalian cells revealed that this protein moves to the cell cortex on vesicles that preferentially contain Rab11, and that Sec8 remains with these vesicles until SNARE mediated fusion at the furrow [54].

Our results also indicate that a common membrane trafficking pathway may link anaphase cell elongation and cytokinesis. Previous studies have shown a fundamental connection between cell size and the extent of anaphase elongation [55], suggesting that limits in cell size and available surface area may dictate the degree to which elongation of the spindle at Anaphase B can occur. Our data also demonstrate that cell volume is conserved throughout anaphase and cytokinesis. This implies that, due to geometric constraints, cell surface area must increase as the cell adopts an elongated shape. Consistent with this, surface area addition fails in cells mutant for *onr* or *fun*, and anaphase cell elongation is also disrupted. A small change in aspect ratio is still observed in *onr* and *fun* mutant spermatocytes, which might indicate that a limited reservoir of excess membrane/elasticity exists in the plasma membrane at the beginning of anaphase elongation. Alternatively, this may result from residual exocyst function in the hypomorphic *onr* and *fun* alleles. Interestingly, previous work has also shown that cells with lengthened chromosomes undergo anaphase elongation to a greater degree, suggesting that there may, in turn, be an instructional cue from the spindle to the elongation machinery [56]. An additional component to anaphase elongation is the contribution of actin-dependent cortical stiffness. Recently, it has been shown that a PP1-Sds22-Moesin pathway is required for cortical polar relaxation and that excess rigidity can inhibit anaphase elongation and spindle function [57, 58]. It therefore appears that exocyst-dependent membrane trafficking may function along with cytoskeletal regulation to direct cell elongation during division.

Initiation of cleavage furrow ingression occurs within a few minutes (6.6±1.1 minutes, n = 8) of the start of anaphase elongation. This tight juxtaposition in time of both anaphase elongation and cytokinesis suggests that these two processes may be poised to take advantage of similar cell shaping and membrane trafficking mechanisms. As discussed above, the requirement for targeted membrane addition during cytokinesis is well-established [3,59,60]. The conservation of volume that we observed throughout our quantitative measurements indicates that, similar to the geometric requirements imposed on anaphase elongation, surface area must increase as the cell divides into two daughter cells. Our data support this approximate 26% predicted total increase in surface area, and illustrate that surface area addition peaks early in cytokinesis, consistent with findings from a study on Arf6 function in spermatocytes [8]. We further observed that this increase in surface area initiated at anaphase elongation and continued as cytokinesis progressed. Surface area addition was disrupted in *onr* and *fun* mutant cells and cytokinesis failed almost immediately on initiation. These results are consistent with a shared requirement for exocyst-dependent trafficking in anaphase cell elongation and cytokinesis. It may also be that essential guidance factors or components of the ingression machinery are dependent on membrane delivery to the cleavage furrow. As Rab11 has been implicated in guiding central spindle function [61], an interesting aspect for future studies will be to further examine the relationship between central spindle function and exocyst-dependent membrane delivery in directing the profound cell shape changes that occur in cell division. It is also intriguing to note that exocyst function is required during plant cytokinesis [62,63,64], suggesting a potentially ancient connection between membrane trafficking pathways and cell division.

## Materials and Methods

### Molecular biology and rescue experiments

A 6,563bp BamHI-XbaI genomic fragment was subcloned from BACR02L23 into pCasper4. Sec8 was the only complete predicted open reading frame in this genomic fragment. Transgenic stocks expressing this transgene were crossed to *fun* ^*z1010*^ and assayed for rescue of cytokinesis defects. To generate the GFP-Exo84 construct, the EGFP coding sequence was fused in frame to the amino terminus of the full-length cDNA corresponding to *Exo84* and cloned into the pCaSpeR4 under the control of α-tubulin promoter (as described in [13]). GFP-Exo84 was crossed into the *onr* background to test for phenotypic rescue of male sterility and cytokinesis failures.

### Fly stocks

Flies were maintained at 25°C by standard procedures. *y w* and Oregon-R were used as wild-type controls. *onr* ^*z4840*^ is synonymous with *onr* ^142-5^ and corresponds to Z4840 in the Zuker viable collection, while *fun* ^*z1010*^ is synonymous with *fun* ^145-27^ and Z1010 in the Zuker collection [4]. Time-lapse imaging was conducted with Sqh-GFP [36], PLCδ-PH-GFP [37], and β-Tub-GFP [38]. *Df(3R)Espl3* and *Df(3R)Exel6145* (Bloomington) uncover *onr* and *fun*, respectively. The *Rab11* ^*E(To11)*^ and *Rab11* ^*93Bi*^ mutant strains were described previously [10]. *Rab11-GFP* was a gift from R.S. Cohen [65]. Strains carrying the *UASp-YFP-Rab11* transgenes [66] were obtained from the Bloomington Drosophila Stock Center. Flies carrying the *UASp-HA-Sec8* transgene were a gift from T.L. Schwarz (Harvard Medical School). Bam-Gal4 [67] was used to drive expression of YFP-Rab11 from the *UASp-YFP-Rab11* transgenes and HA-Sec8 from the *UASp*::*HA-Sec8* transgene.

### Immunofluorescence staining and microscopy

Cytological preparations were made with testes from third instar larvae or adults. To visualize GFP-Exo84 or Rab11-GFP, larval testes were fixed in 4% methanol-free formaldehyde (Polysciences, Warrington, PA), as previously described [7]. Following fixation, testes were incubated with GFP-Booster (ChromoTek) diluted 1:100 in phosphate-buffered-saline (PBS), as described in [14]. To visualize α-Tubulin and either Sec8 or Sec5, larval testes were dissected in PBS (Sigma-Aldrich) and transferred into a drop (4 μl) of PBS containing 4% methanol-free formaldehyde placed on a coverslip. Preparations were kept at room temperature for two minutes before gently squashing on an inverted slide. They were then fixed for an additional 5 minutes before immersing in liquid nitrogen. After removing the coverslip, preparations were immersed in PBS for five minutes and permeabilized in PBS containing 0.1% Triton-X (PBT) for 10 minutes at room temperature and washed in PBS 0.1% Tween-20 for 20 minutes before incubation with primary antibodies diluted in PBT containing 3% BSA. To visualize α-Tubulin and Sec8 of cells shown in S2 Fig, larval testes were dissected in 0.7% NaCl and transferred into a drop of PBS containing 0.5% Triton for two minutes. Testes were then transferred to 4 μl of PBS containing 3.7% formaldehyde on a coverslip, gently squashed on an inverted slide and fixed for ten minutes before immersing in liquid nitrogen. After removing of the coverslip, samples were immersed for 20 minutes in cold methanol (-20°C) and in PBS containing 0.1% Triton for 20 minutes at room temperature. For immunostaining with other antibodies, preparations were fixed using 3.7% formaldehyde in PBS and then squashed in 60% acetic acid as previously described [10]. Monoclonal antibodies were used to stain α-Tubulin (1:300; Sigma-Aldrich, T6199) and Sec5 (1:30; [25]), gift from T.L. Schwarz (Harvard Medical School). Polyclonal antibodies were as follows: rabbit anti-Lva (1:500; [40]), gift from O. Papoulas (University of Texas at Austin); rabbit anti-Gio (1:2000; [41]), guinea pig anti-Sec8 (1:250; [51]), gift from Ulrich Tepass (University of Toronto); rat anti-Rab11 (1:200; [65]), gift from R.S. Cohen; rabbit anti-anillin (1:1000; this study). Secondary antibodies were Alexa 555-conjugated anti-rabbit IgG (1:300, Life Technology), FITC-conjugated anti-mouse/anti-rat IgG (1:20, Jackson ImmunoResearch), Alexa 555-conjugated anti-guinea pig IgG (1:300, Life Technology). All incubations with primary antibodies (diluted in PBT containing 3% BSA) were performed at 4°C overnight. Incubations with secondary antibodies were performed at room temperature for 50 minutes. After immunostaining, all preparations were mounted in Vectashield mounting medium with DAPI (Vector Laboratories) to stain DNA and prevent photobleaching. Images were captured with a charged-coupled device (CCD camera, Photometrics Coolsnap HQ), connected to a Zeiss Axioplan epifluorescence microscope equipped with an HBO 100-W mercury lamp and 40X and 100X objectives. The number of Golgi stacks per cell was calculated manually, by analyzing images of G2 spermatocytes at S5 stage stained for Tubulin, Lva and DNA. The size of Golgi bodies was measured using Image J software (NIH; http://rsbweb.nih.gov/ij/) by manual demarcation with a limiting polygon and calculation of its area (see also [13] for the procedure).

### Confocal microscopy and time-lapse imaging

Time-lapse imaging of PLCδd-PH-GFP and β-Tub-GFP was performed on a spinning disk confocal microscope from Zeiss and Solamere Technologies Group with 63x/1.4NA objectives. Germline cells were imaged after dissection and placement in Voltalef 10S oil. Live imaging was performed using exposure settings of 250 msec and 4D image sets were acquired every 60 seconds with a Z-step of 1 micron. Images were edited using Adobe Photoshop.

Larval testes expressing Sqh-GFP were dissected and prepared for time lapse using the protocol described previously [13]. Meiotic divisions were analyzed with a Zeiss Axiovert 20 microscope equipped with a 63X, 1. 25 NA objective and a filter wheel combination (Chroma Technology Corp.). Images were collected at 1-minute time intervals with a CoolSnap HQ camera (Photometrics) controlled by MetaMorph software (Universal imaging). Eleven fluorescent optical sections were captured at 1 μm Z-steps and maximally projected using MetaMorph software.

### Image segmentation

We performed a 3D seeded watershed algorithm using the MATLAB image processing toolbox. For the first frame of each movie, we manually initialized the seeds separately in each Z-layer to construct a single 3D seed (see S4A–S4D Fig); using the seed, the 3D watershed algorithm was applied on the 3D Gaussian filtered image stack (σ_x_ = σ_y_ = 1 pixel = 0.166 μm, σ_z_ = 0.2 pixels = 0.2 μm). For each subsequent frame, the new seeds were then generated automatically by eroding the results of the watershed segmentation from the last frame, with occasional manual intervention, e.g., to ensure that the seeds masked off any bright features inside the cell, such as spindles.

### Aspect ratio

We defined the aspect ratio as the length of the major axis divided by the length of the minor axis (see S4E Fig). We determined the major axis length in 3D by finding the maximum distance between any pair of surface positions of the cell. As the minor axis length, we used the diameter of the larger sphere-like lobe of the cell, which we computed through a 3D distance transform on the 3D binary image of the cell. With these definitions for the major and minor axis length, a perfect sphere will have an aspect ratio of 1, and two just-touching spheres of equal radius will have an aspect ratio of 2. Since cells are frequently ‘deformed’ due to mechanical contact with neighboring cells, they generally don’t approximate perfect spheres, so that aspect ratios are frequently >1 before the initiation of division, and can reach values >2 during division.

### Convex hull volume ratio

The convex hull of a cell is the smallest convex volume that fully contains the segmented volume of the cell on the inside (see S4F Fig), i.e. it represents the segmented cell volume with all the concave regions next to the cleavage furrow ‘filled in’. We defined the convex hull volume ratio (CHVR) as the convex hull volume of the cell divided by the actual segmentation volume of the cell, which is thus a volume-based measure of furrow ingression. For a cell without concavities, the CHVR will be equal to one; conversely, when concavities are present, the CHVR will increase with the relative volume of the concave areas. Thus, a CHVR value of 1.1 means that the volume of the concavity is equal to 10% of the segmented cell volume. For reference, the CHVR of two touching spheres of equal radius (an idealization of two daughter cells in contact after division) is 1.25.

### Volume, surface area, aspect ratio, and CHVR alignment

In order to average time-courses of multiple experiments for a given condition, and to effectively compare wild-type, *onr*, and *fun* conditions with each other, cell shape measurements have to be aligned to a common ‘reference’ time point that represents the initiation of cytokinesis. While the *onr* and *fun* mutants do not undergo significant rate changes in volume, surface area, or CHVR that could provide useful fiduciary markers for temporal alignment, we observed that the mutants still undergo a distinct initial increase of their aspect ratio–i.e., they show a small but significant elongation, even in the absence of effective furrow ingression. We used an automated algorithm to identify this ‘shoulder’ point of the aspect ratio in each individual cell trace (see samples in see S4G Fig), and used it as a reference time point (representing t = 0) for subsequent temporal alignment. Mathematically, the reference time point is the first time point at which the slope of the forward 10 min time window increases by 20% (wild type) or 60% (mutants) relative to the backward 10 min time window. This automated alignment was in excellent agreement with manual alignment. A similar inflection point in CHVR was used to determine the start of cytokinesis (arrowhead marker in Fig 4G–4J).

### Transmission electron microscopy

Testes for transmission electron microscopy were prepared using a protocol modified from [68]. Briefly, testes from third instar larvae and 0–3 day-old adults were dissected in ice-cold phosphate buffer (PB) (pH = 7.4) and immediately transferred into ice-cold Trump’s fixative, where they were kept for 2h. Samples were post-fixed with 1% OsO4 for 1 hour, rinsed and dehydrated with an acetone series and embedded in Quetol-Spurr or Epon resin. Images were acquired with a JEOL JTE141011 (JEOL, Peabody, MA; The Hospital for Sick Children Electron Microscopy Facility) and were processed with Adobe Photoshop.

### Protein expression and purification, antibody generation

GST-full-length *Drosophila* Rab11 was expressed in BL21-CodonPlus [DE3] cells (Invitrogen) and purified using HiTrap affinity columns (GSTtrap FF, and GSTtrap HP columns, GE Healthcare) operated with AKTA 900 Fast Protein Liquid Chromatography as previously described [13]. Polyclonal antisera were raised against the purified GST-Rab11 protein. Polyclonal anti-anillin antibodies were raised against the N-terminal 270 amino acids of anillin, following the procedure described in [69]. Immunization was carried out at Agro-Bio Services (www.agro-bio.com) using standard procedures. The anti-GST-Rab11 and anti-anillin antisera were depleted of anti-GST antibodies and affinity-purified against either GST-Rab11 or GST-anillin before use in immunoblotting.

### Western blotting and immunoprecipitation

Co-IP experiments from testes expressing GFP- or YFP-tagged proteins were performed using GFP Trap-A kits beads purchased from ChromoTek (Planegg-Martinsried), as previously described [12]. For the experiment in Fig 9D, testes expressing GFP-Exo84 and HA-Sec8 or HA-Sec8 alone were used as controls. Samples were separated on Mini-PROTEAN TGX precast gels (Bio-Rad) and blotted to PVDF membranes (Bio-Rad). Membranes were blocked in Tris-buffered saline (Sigma-Aldrich) with 0.05% Tween-20 (TBST) containing 5% nonfat dry milk (Bio-Rad; Blotting GradeBlocker) for 3–4 hours at room temperature followed by incubation with primary and secondary antibodies diluted in TBST. Primary antibodies used for immunoblotting were as follows: rat monoclonal anti-GFP, (3H9; 1:1000; ChromoTek), mouse anti-Rab11 (1:1000; this study), rat anti-HA (1:1000; Roche). HRP-conjugated secondary antibodies (GE Healthcare) were used at 1:5000. After incubation with the antibodies, blots were washed in TBST and imaged using an ECL detection kit (GE Healthcare).

## Supporting Information

S1 Fig Alignment of *Drosophila* Sec8 protein with human and mouse Sec8 homologs. (*) fully conserved residue; (:) conservation between groups of strongly similar properties; (.) conservation between groups of weakly similar properties. The site of the conserved Serine residue at position 322 is outlined in red, which is mutated to a Phenylalanine in *fun* ^*z1010*^.
(JPG)

S2 Fig Localization of Sec8 protein in dividing spermatocytes. Testes were fixed with formaldehyde and methanol as per [13] and stained for Sec8 (red), Tubulin (green) and DNA (blue). Arrows and Arrowheads indicate the cortical Sec8 accumulation. Scale bar, 10 μm.
(TIF)

S3 Fig Localization of GFP-Exo84 in wild type spermatocytes. Testes expressing GFP-Exo84 were fixed and incubated with GFP-Booster and stained for DNA. Left panel, Primary spermatocyte at G2; Right panel, Telophases II. Scale bar, 10 μm.
(TIF)

S4 Fig Automated segmentation and analysis of dividing spermatocytes. (A) Raw image of a cell undergoing cytokinesis (cross section near ‘equator’ of the cell). (B) 3D Gaussian filtered image. (C) Red overlaid regions represent watershed seeds applied to the image. (D) Final watershed segmentation lines of this z-layer overlaid on the image. (E) *Aspect Ratio*: The aspect ratio is the length of the long axis (D_1_) divided by the short axis (D_2_), where D_2_ is the maximum diameter of the larger ‘lobe’. (F) *Convex hull*: The convex hull is the smallest convex volume that contains the (potentially concave) segmented cell volume. (G,H) 3D segmentation of dividing cell (G) as compared to convex hull volume (H). The convex hull and 3D segmentation data are identical prior to cell division (left images) and during anaphase elongation (middle images), but diverge during furrow ingression/cytokinesis. (I) *Data alignment*: Sample traces of aspect ratio time courses. The ‘shoulder’ of the observed increase of the Aspect ratio (small red circles)–which is determined computationally through the increase in slope–serves as a ‘reference’ time point which is used as t = 0 for subsequent data alignment.
(TIF)

S1 Movie Wild type primary spermatocytes, expressing the myosin light chain fusion protein Sqh-GFP undergoing anaphase and telophase. (MOV)

S2 Movie Primary spermatocyte from *onr* males, expressing the myosin light chain fusion protein Sqh-GFP undergoing anaphase and telophase. (MOV)

S3 Movie Primary spermatocyte from *fun* males, expressing the myosin light chain fusion protein Sqh-GFP undergoing anaphase and telophase. (MOV)

S4 Movie Wild type cell division in male germline cells expressing PLCδd-PH-GFP and β-Tub-GFP. 4D image sets were acquired every 60 seconds with a Z-layer spacing of 1 micron. Scale bar is 10 μm.
(MOV)

S5 Movie Cell division in an *onr* mutant male germline cell expressing PLCδd-PH:GFP and β-Tub:GFP. 4D image sets were acquired every 60 seconds with a Z-layer spacing of 1 micron. Scale bar is 10 μm.
(MOV)

S6 Movie Cell division in a *fun* mutant male germline cell expressing PLCδd-PH:GFP and β-Tub:GFP. 4D image sets were acquired every 60 seconds with a Z-layer spacing of 1 micron. Scale bar is 10 μm.
(MOV)
