# Supplementary figures and images for: Exocyst-Dependent Membrane Addition Is Required for Anaphase Cell Elongation and Cytokinesis in Drosophila
Source: PLoS Genet. 2015 Nov 3;11(11):e1005632. doi: 10.1371/journal.pgen.1005632 (PMC4631508; doi:10.1371/journal.pgen.1005632)

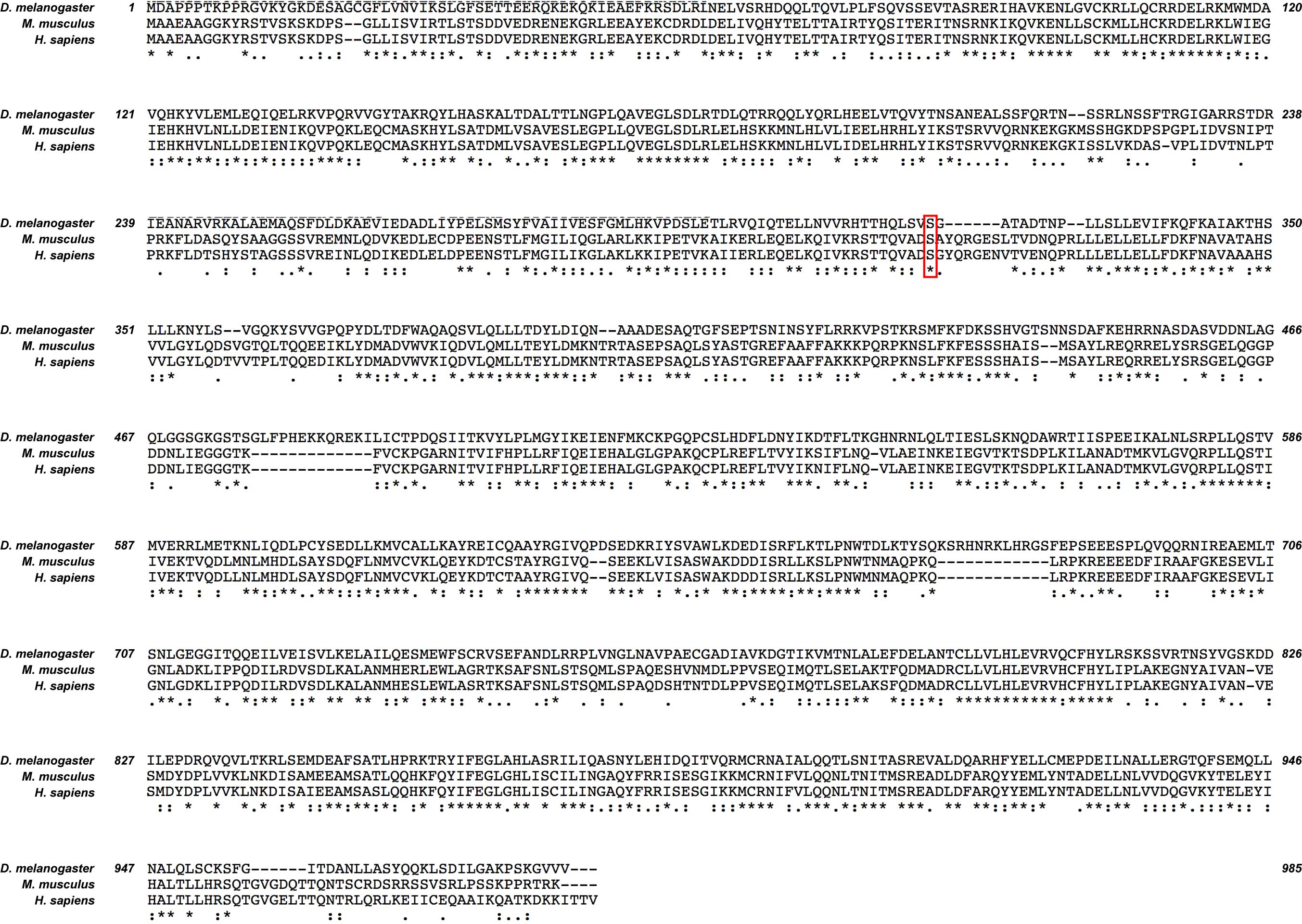

Supplement: S1 Fig — (*) fully conserved residue; (:) conservation between groups of strongly similar properties; (.) conservation between groups of weakly similar properties. The site of the conserved Serine residue at position 322 is outlined in red, which is mutated to a Phenylalanine in fun z1010. (JPG) [file pgen.1005632.s001.jpg]

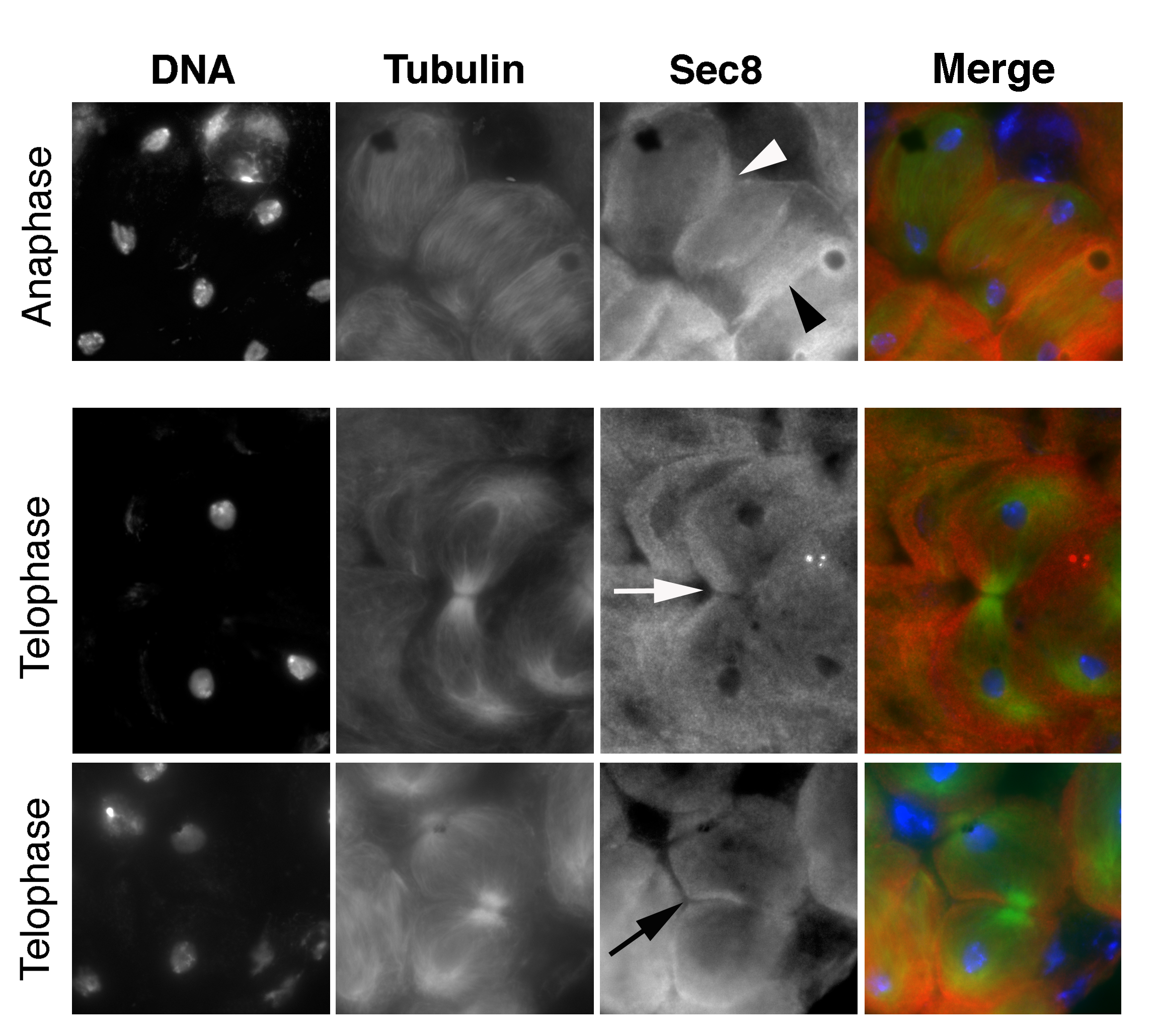

Supplement: S2 Fig — Testes were fixed with formaldehyde and methanol as per [13] and stained for Sec8 (red), Tubulin (green) and DNA (blue). Arrows and Arrowheads indicate the cortical Sec8 accumulation. Scale bar, 10 μm. (TIF) [file pgen.1005632.s002.tif]

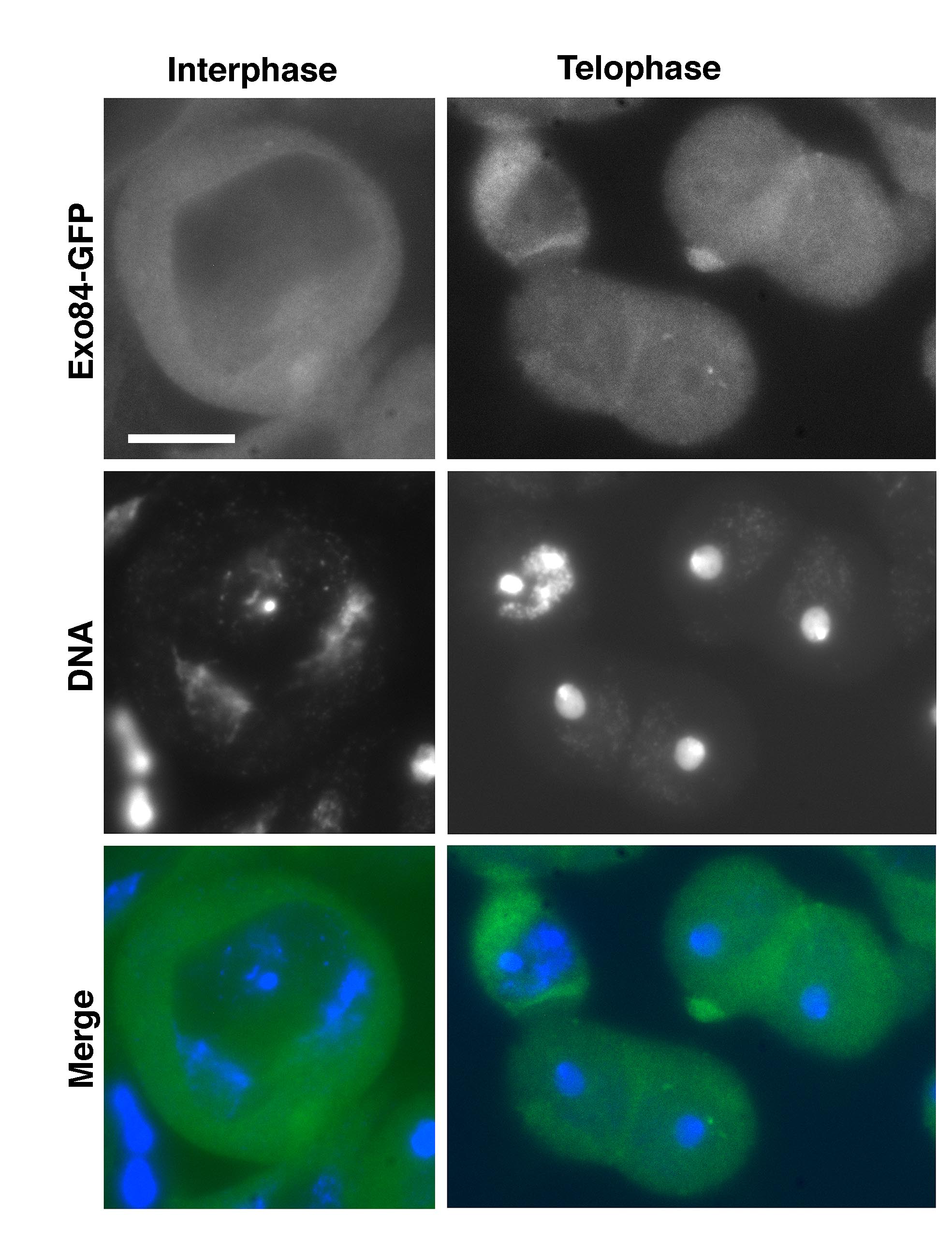

Supplement: S3 Fig — Testes expressing GFP-Exo84 were fixed and incubated with GFP-Booster and stained for DNA. Left panel, Primary spermatocyte at G2; Right panel, Telophases II. Scale bar, 10 μm. (TIF) [file pgen.1005632.s003.tif]

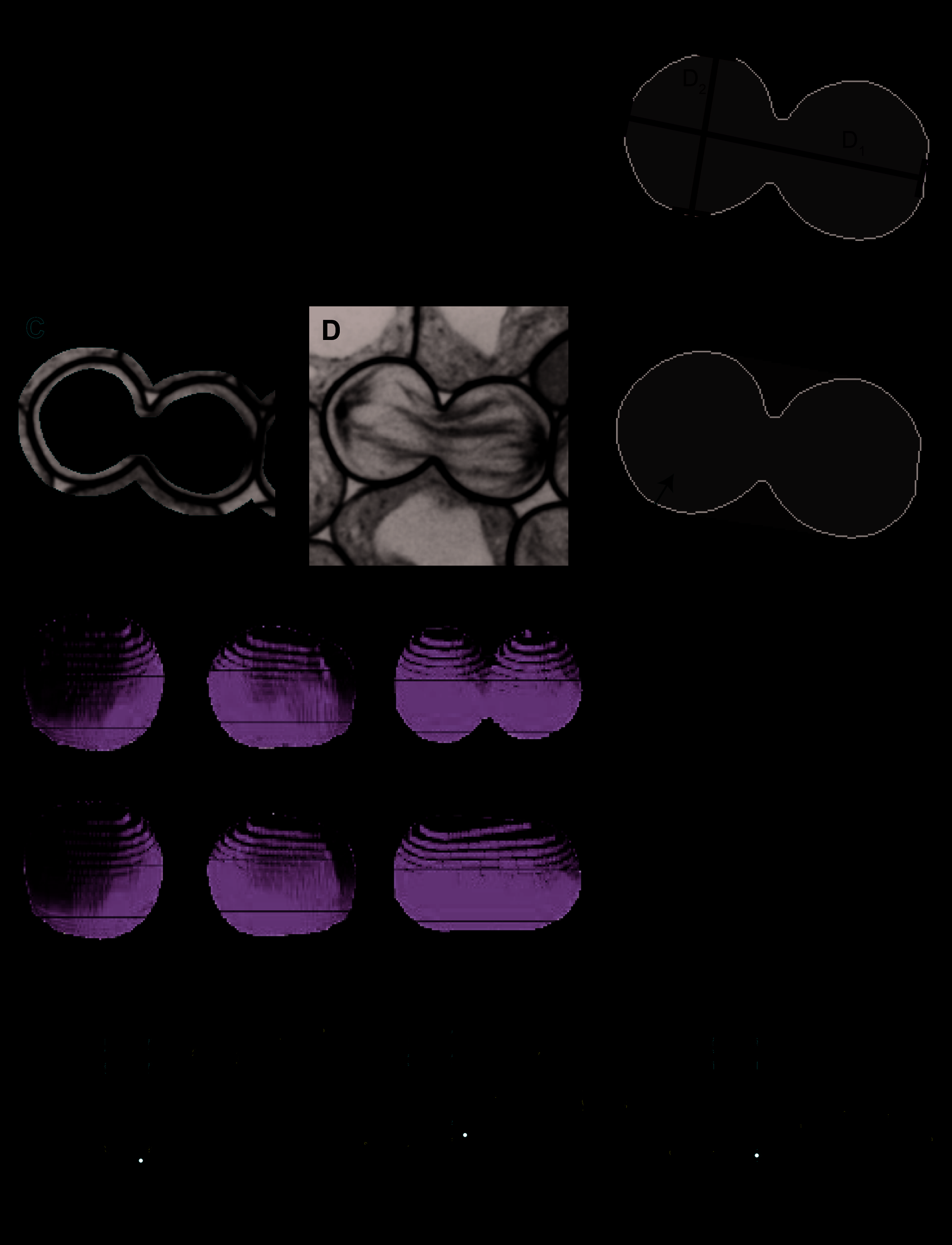

Supplement: S4 Fig — (A) Raw image of a cell undergoing cytokinesis (cross section near ‘equator’ of the cell). (B) 3D Gaussian filtered image. (C) Red overlaid regions represent watershed seeds applied to the image. (D) Final watershed segmentation lines of this z-layer overlaid on the image. (E) Aspect Ratio: The aspect ratio is the length of the long axis (D1) divided by the short axis (D2), where D2 is the maximum diameter of the larger ‘lobe’. (F) Convex hull: The convex hull is the smallest convex volume that contains the (potentially concave) segmented cell volume. (G,H) 3D segmentation of dividing cell (G) as compared to convex hull volume (H). The convex hull and 3D segmentation data are identical prior to cell division (left images) and during anaphase elongation (middle images), but diverge during furrow ingression/cytokinesis. (I) Data alignment: Sample traces of aspect ratio time courses. The ‘shoulder’ of the observed increase of the Aspect ratio (small red circles)–which is determined computationally through the increase in slope–serves as a ‘reference’ time point which is used as t = 0 for subsequent data alignment. (TIF) [file pgen.1005632.s004.tif]
